# Supplementary figures and images for: A network pharmacology approach to decipher the total flavonoid extract of Dracocephalum Moldavica L. in the treatment of cerebral ischemia- reperfusion injury
Source: PLoS One. 2023 Jul 26;18(7):e0289118. doi: 10.1371/journal.pone.0289118 (PMC10374249; doi:10.1371/journal.pone.0289118)

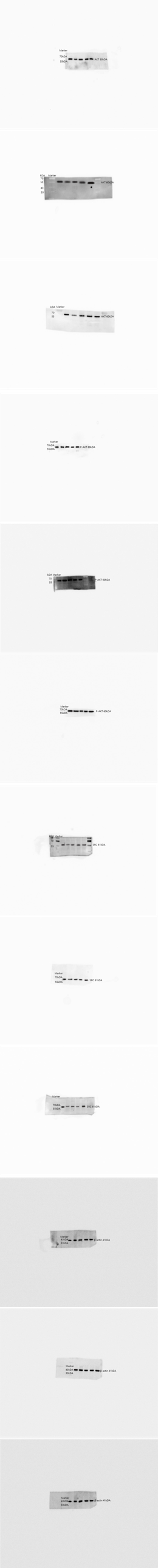

Supplement: S1 Raw images — (TIF) [file pone.0289118.s001.tif]
