# Supplementary material for: A network pharmacology approach to decipher the total flavonoid extract of Dracocephalum Moldavica L. in the treatment of cerebral ischemia- reperfusion injury
Source: PLoS One. 2023 Jul 26;18(7):e0289118. doi: 10.1371/journal.pone.0289118 (PMC10374249; doi:10.1371/journal.pone.0289118)
Supplement: Attachment — Submitted filename: Response to Reviewers.docx [file pone.0289118.s002.docx]

Dear editors and peer reviewers,

First of all, it's a great honor to get your valuable suggestions. I attach great importance to your review conclusion and will revise it according to your suggestions to make the article more complete and rigorous. The following are my answers to your Questions, hoping to get your approval.

Responses to general guidelines

1. Edited manuscript to meet PLOS ONE's style requirements, including the file name.

2. Added (1) methods of sacrifice, (2) methods of anesthesia and analgesia.

3. We moved the ethics statement to the Methods section of the manuscript line 176. And improved the relevant contents of the ethics statement, including "All animal experiments(Approval number: XJIMM-20211006) were reviewed and approved by the Ethics Committee of the Xinjiang Institute of Materia Medica. Animal welfare was monitored daily by animal care staff. The approval document (Ethics Statements) has been uploaded to other Item. Human participants and/or tissues were not involved in this study."

4. Provided the original underlying images for Western blot analysis as Supporting Information files at the end of manuscript, and the citations in the manuscript are updated accordingly.

5. We checked the reference list of the manuscript to ensure that it was complete and correct.

Thank you for considering our revised manuscript for publication. We look forward to any additional comments or questions.

Yours sincerely,

Xu Hu

**Reviewer #1:**

Dear editors and peer reviewers,

First of all, it's a great honor to get your valuable suggestions. I attach great importance to your review conclusion and will revise it according to your suggestions to make the article more complete and rigorous. The following are my answers to your Questions, hoping to get your approval.

Question 1. Why only male rats were chosen for the study?

Answer: Studies have shown that the physiological structure of female mammals is more complex than that of male mammals, which is subject to certain uncertainties such as pregnancy and estrous cycle, which may increase the experimental uncertainty. Besides, this experiment involves indicators of inflammation. Female rats have different sensitivities to inflammation. In conclusion, male rats were selected for this study.

Question 2. Lot or CAT numbers for the kits and antibodies used throughout the study were not mentioned, although it’s crucial for such a study.

Answer: Corresponding changes have been made in the revised manuscript line 162.

SDS-PAGE gel preparation kit (P0012A, Beyotime Biotechnology Company, China ), lactate dehydrogenase (LDH) release assay kit (A020-2-2, Nanjing Jiancheng Institute of Bioengineering, China), Nitric Oxide (NO) assay kit(A013-2-1, Nanjing Jiancheng Institute of Bioengineering, China), Malondialdehyde (MDA) assay kit (A003-4-1, Nanjing Jiancheng Institute of Bioengineering, China), IL-1β ELISA kit (E-EL-R0012c, Elabscience Biotechnology, China), IL-6 ELISA kit (E-EL-R0015c, Elabscience Biotechnology, China), TNF-α ELISA kit (E-EL-R2856c, Elabscience Biotechnology, China), BCA protein quantification kit (23225, Thermo Fisher Pierce, US), primary antibodies AKT(#4060, CST Company, US), p-AKT(#9272, CST Company, US), SRC(ab40660, Abcam, US), β-actin (ab8226, Abcam, US), GAPDH antibody(TA-08, Zhongshan Jinqiao Company, China), horseradish enzyme-labeled goat anti-mouse IgG (ZB-2305, Zhongshan Jinqiao Company, China), horseradish enzyme-labeled goat anti-rabbit IgG (ZB-2301, Zhongshan Jinqiao Company, China)

Question 3. After blood collection and centrifugation, how was the supernatant stored? Also, how would the storage conditions affect LDH, MDA and NO levels?

Answer: After centrifugation, the supernatant was divided into several EP tubes and stored in the refrigerator at -20℃. Before use, it should be melted at 4℃ and then used. In order to ensure the accuracy and reliability of the determination results of LDH, MDA and NO, samples should be kept at low temperature, sealed and neutral conditions for determination as soon as possible. Our sample storage conditions can meet the requirements.

Question 4. What kind of effect is being validated by administering TFDM before the surgery not just after the surgery?

Answer: In this research, we use preventive medication. The purpose of preventive administration is to observe the initial efficacy. We consider that if even preventive administration can't see the efficacy, there is no need to conduct an investigation on therapeutic administration.

Question 5. What was the success rate of establishing the CIRI rat model? As it’s known such invasive procedure has a mortality rate. Were all the rats assigned from the beginning alive throughout the rest of the study?

Answer: After the training of model establishment, before the experiment, the modeling rate of CIRI rat model was about 70%. Because after the rat model is made, it will cause certain death due to anesthesia, arterial rupture, cerebral hemorrhage and other reasons, and the surviving rats are all alive before the subsequent blood collection and execution.

Question 6. As stated in the study, TFDM shows promise as a therapeutic strategy for CIRI. Would that indicate the use of TFDM as a prophylactic in general prior to the condition or after the ischemic injury?

Answer: At present, TFDM can be considered as a candidate drug for preclinical development. After completing the systematic preclinical research, we can enter the clinical stage research, which needs to be tested on human body, so the drug must be approved by the national drug supervision and administration department. In China, research and development institutions of new drugs need to submit clinical applications for new drugs to the National Medical Products Administration (NMPA), and only after obtaining permission can they conduct human clinical trials. Clinical research is divided into four stages: phase I clinical trial, phase II clinical trial, phase III clinical trial and phase IV clinical research (post-marketing monitoring of drugs). After completing the clinical trials in the above three stages, the clinical data are statistically analyzed, which proves that the drug is safe and effective, and at the same time, the drug quality is controllable, that is, the three points of safety, effectiveness and controllability meet the requirements, and the drug marketing license holder submits a new drug marketing application to the drug supervision department. After obtaining the approval of the drug supervision department, the drug can be marketed for doctors and patients to choose.

Thank you for considering our revised manuscript for publication. We look forward to any additional comments or questions.

Yours sincerely,

Xu Hu

**Reviewer #2:**

Dear editors and peer reviewers,

First of all, it's a great honor to get your valuable suggestions. I attach great importance to your review conclusion and will revise it according to your suggestions to make the article more complete and rigorous. The following are my answers to your Question, hoping to get your approval.

Question 1. Animal ethical Committee number should be mentioned in the manuscript.

Answer: It has been revised in the revised manuscript line 176, and the approval number is XJIMM-20211006.

Question 2. The grouping of animals and drug treatment pattern is not clear.

Answer: It has been revised in the revised manuscript line 179. After the rats are bought back, they are kept in the animal house for 7 days to adapt to the environment. After the rats adapt to the environment, the SD rats were randomly divided into a sham operation group, a model group, and TFDM 30, 60 and 120 mg groups, a total of 5 groups, 10 rats in each group.The rats of the three TFDM dose groups were intragastrically administered with TFDM 30, 60 and 120 mg·kg^-1^ per day 7 d before surgery, respectively. The rats of the sham operation group and the model group were intragastrically administered with corresponding doses of normal saline, it lasted for 7 days, on the morning of the eighth day, the CIRI model was established two hours after the administration was completed, 45min after the operation caused cerebral ischemia, and 24 hours after reperfusion, it created a cerebral ischemia-reperfusion injury model, and the samples were taken 24 hours after operation.

Question 3. I note that there have been similar research works published in the past. To address this issue, I recommend that you provide a clear explanation of how your current research builds upon and/or differs from the existing literature.

Answer: Our research group comprehensively verified the prediction results based on bioinformatics prediction data and combined with biological experiments to avoid false positive or false negative results caused by relying entirely on "bioinformatics". Combining computer virtual data with real pharmacological experimental data to reveal the mechanism of TFDM in treating cerebral ischemia-reperfusion injury comprehensively, accurately and efficiently is one of the innovations of this topic. At the same time, we screened the active components in TFDM by molecular docking with key targets. It provides a preliminary basis for promoting the separation of chemical components of natural drugs and their application in the treatment of brain diseases.

Question 4. Some of the references are not as per the journal guidelines

Answer: Corresponding changes have been made in the revised manuscript References section.

Thank you for considering our revised manuscript for publication. We look forward to any additional comments or questions.

Yours sincerely,

Xu Hu

**Reviewer #3:**

Dear editors and peer reviewers,

First of all, it's a great honor to get your valuable suggestions. I attach great importance to your review conclusion and will revise it according to your suggestions to make the article more complete and rigorous. The following are my answers to your Question, hoping to get your approval.

Question 1. In Abstract section line 18, authors should replace “Disease” with “injury” as cerebral ischemia- reperfusion injury (CIRI) is usually caused by caused by ischemic stroke seriously or restriction of blood supply. Line 28, 90, in vitro should be written in Italic. Abstract is lack of key findings of the study.

Answer: Corresponding changes have been made in the revised manuscript Abstract section.

Some additional molecular docking results showed that acacetin-7-O (- 6''-acetyl) -glucopyranoside, acacetin7-O-β-D-glucopyranoside, apigenin-7-O-β-D-galactoside respectively had good affinity for SRC protein. acacetin-7-O (- 6''-acetyl) -glucopyranoside,

acacetin-7-O-β-D-glucuronide, acacetin7-O-β-D-glucopyranoside had good affinity for AKT1 protein, respectively. acacetin-7-O (- 6''-acetyl) -glucopyranoside is expected to be used as a candidate monomer component for natural drugs for further development.

Question 2. The ethical approval number should be added in methodology section as authors mentioned the study has received approval from the Ethics Committee of the Xinjiang Institute of Materia Medica.

Answer: It has been revised in the revised manuscript line 176, and the approval number is XJIMM-20211006.

Question 3. Safety profile of total flavonoid extract of *Dracocephalum Moldavica* L. should be addressed.

Answer: This research group investigated the safety of TFDM from the following two aspects:

1) To investigate the acute toxicity and death of TFDM after one-time administration to mice and multiple times in a day.
2) To investigate the toxic reaction of TFDM induced by gavage for consecutive 25 weeks, including the first symptom and severity, target organ of toxic reaction and its recovery and development.

Results 1) showed that TFDM given to mice by gavage with the maximum dose of 19.2g/kg.d (equivalent to 711 times of the proposed clinical dose) in a day did not produce significant acute toxicity during the specified observation period. It indicated that this preparation had no obvious acute toxicity and its medication was safe.

Results 2) Compared with the control group, no abnormal changes related to drug administration were observed in rats' body weight, organ coefficient, hematology, and blood biochemistry indexes. Histological examination also revealed no pathological changes related to drug effects; It was considered that TFDM had no obvious toxicity to rats when given by gavage within the specified dose and time limit.

Question 4. Please add software specifications used in statistical analysis (Graph prism). More robust & appropriate statistical analysis is also required.

Answer: Statistical analysis was performed with GraphPad Prism 8.0 software Version8.0.2(263). All data were expressed as the mean ± standard deviation (SD). The differences among multiple groups were evaluated using the one-way analysis of variance (ANOVA). The difference between the means was considered statistically significant at P < 0.05.

Question 5. Limitation of the study should be discussed in limitation section of the manuscript.

Answer: Corresponding changes have been made in the revised manuscript line 413.

One of the shortcomings of traditional Chinese medicine in treating diseases is its slow onset, and the rapid onset of chemical drugs is its advantage over traditional Chinese medicine. Therefore, the rational combination of traditional Chinese medicine and chemical drugs can be considered at present, which provides a new direction for anti-CIRI research.

In the future, we will conduct further research on the active components in TFDM that have strong binding affinity with key targets.We believe that with the deepening of the research on the pathogenesis of CIRI, the research on the mechanism of action, treatment time window and pharmacokinetics of TFDM and its compound anti-CIRI is becoming increasingly clear, and the current situation of CIRI treatment difficulties will certainly make a breakthrough.

Question 6. Authors should describe the Future perspective and clinical significance of the study.

Answer: Corresponding changes have been made in the revised manuscript line 354.

Cerebral ischemia-reperfusion injury(CIRI) is a common and frequently-occurring disease in the clinic. The mortality and disability rate are extremely high. It seriously threatens human health and life, and the burden on society and families is immeasurable. Therefore, scientists and clinicians need to make great efforts to find potential pathogenic mechanisms, so as to take appropriate preventive measures and successful treatment methods. CIRI involves a variety of mechanisms, including free radical action, excitatory amino acids, cellular Ca^2+^ overload. To understand the mechanism of CIRI and deepen the understanding of the process of cerebral ischemic injury is conducive to the research and selection of medication. so as to achieve the purpose of treatment. Natural medicines have a long history of treatment for cerebral ischemia and have accumulated rich theoretical knowledge. Screening active ingredients from natural medicines is also the current trend of research and development. The whole world is promoting the separation of chemical components of natural medicines and their application in the treatment of brain diseases. Our research group has continuously conducted a series of studies on this issue, and have made great progress. This study found that TFDM has unique advantages and potential in the treatment of CIRI because of its diverse chemical components and structures, and can intervene the pathological process of CIRI through multiple targets and channels as a whole. In this paper, TFDM pharmacodynamics and its mechanism of action are taken as the breakthrough point, in order to provide theoretical basis for drug research and development of CIRI and scientific basis for in-depth research on prevention and treatment of CIRI in traditional Chinese medicine.

Question 7. Authors should add abbreviations list in the manuscript.

Answer: Corresponding changes have been made in the revised manuscript.

cerebral ischemia-reperfusion injury (CIRI)

total flavonoid extract of *Dracocephalum moldavica* L. (TFDM)

recombinant tissue plasminogen activator (rtPA)

Traditional Chinese Medicine (TCM)

*Dracocephalum Moldavica* L. (DML)

effective parts of dracocephalum moldavica (EPDM)

protein-protein interaction (PPI)

Gene ontology (GO)

Kyoto Encyclopedia of Genes and Genomes (KEGG)

molecular function (MF)

biological process (BP)

cellular components (CC)

horseradish peroxidase (HRP)

analysis of variance (ANOVA)

standard deviation (SD)

radio immuno precipitation assay (RIPA)

phenyl methyl sulfonyl fluoride (PMSF)

lactate dehydrogenase (LDH)

Malondialdehyde (MDA)

Thank you for considering our revised manuscript for publication. We look forward to any additional comments or questions.

Yours sincerely,

Xu Hu
